# Cross sectional analysis of the association between mode of school transportation and physical fitness in children and adolescents

**DOI:** 10.1186/1479-5868-10-91

**Published:** 2013-07-17

**Authors:** Lars Østergaard, Elin Kolle, Jostein Steene-Johannessen, Sigmund A Anderssen, Lars Bo Andersen

**Affiliations:** 1 Center for Research in Childhood Health, Institute of Sports Science and Clinical Biomechanics, University of Southern Denmark, Odense 5230, Denmark; 2 Department of Sports Medicine, Norwegian School of Sport Sciences, Oslo 0806, Norway; 3 Faculty of Teacher Education and Sports, SognogFjordane University College, Sogndal 6851, Norway

**Keywords:** Walking, Cycling, Commuting, Transport, Adiposity, BMI

## Abstract

**Objective:**

To investigate the associations between body composition, cardiorespiratory and muscular fitness in relation to travel mode to school in children and adolescents.

**Method:**

Children and adolescents from 40 elementary schools and 23 high schools representing all regions in Norway were invited to participate in the study. Anthropometry, cardiorespiratory and muscular fitness were tested at the school location. Questionnaires were used in order to register mode of transport to school, age, gender and levels of leisure time physical activity.

**Results:**

A total of 1694 (i.e. 60% of all invited participants) children and adolescents at a mean age of 9.6 and 15.6 respectively (SD = 0.4 for both groups) were analyzed for associations with physical fitness variables. Males cycling to school had lower sum of skin folds than adolescents walking to school. Higher cardiorespiratory fitness in adolescents and male cyclists compared to walkers and passive commuters were observed. Among children, cycling and walking to school, higher isometric muscle endurance in the back extensors compared to passive commuters was observed.

**Conclusion:**

Based on this national representative cross-sectional examination of randomly selected children and adolescents there is evidence that active commuting, especially cycling, is associated with a favourable body composition and better cardiorespiratory and muscular fitness as compared to passive commuting.

## Background

The consensus that regular daily physical activity is important for health is underpinned by the national and international health authorities recommendations that children and adolescents should accumulate at least 60 minutes of daily moderate to vigorous intensive physical activity [1-3].

Active transportation to school is a source of physical activity that potentially benefits health and physical fitness. A difference in physical fitness between passive and active commuters could either be due to the direct effect of school transport per se or it could be caused by engagement in more general physical activity which previously has been suggested [4-7].

Regardless of a potential spill over effect it seems plausible that active commuting to and from school could be associated with improved physical fitness. Based on previous studies, however, the adaptive responses to cycling to school seem superior to those from walking. Cycling but not walking, has been associated with 5% [8] and 9% [9] higher levels of maximal oxygen uptake compared to those who are passive commuters. With regards to body mass index (BMI), compared to passive commuters lower BMI has been found in cyclists but not in walkers [10].

A potential greater effect of cycling could be attributed to higher intensity during cycling compared to walking, which has been observed in adults [11] and/or by a longer travelling duration. Regarding walking, the unusual large proportion of cyclists in the studies including Danish samples [8-10] could have compromised the effect estimates of walking from the estimates in passive commuters, due to low statistical power among walkers.

The previous studies investigating the potential physical adaptations to active commuting has usually focused on maximal oxygen uptake and weight status. It is, however, likely that cycling or walking to school also has the potential to affect muscular properties in the core and the lower extremities. Thus, in this study it was hypothesized that not only cardiorespiratory fitness (CRF) and body composition but also other measures of physical fitness such as muscular strength and endurance would be associated to the distinct commuter mode. These expected associations have, however, to date not yet been investigated in a large sample with a high proportion of walkers.

Thus, the purpose of the present study was, in a large national representative sample, composed of numerous walkers and cyclists to investigate the associations between body composition, cardiorespiratory and muscular fitness in relation to travel mode to school.

## Methods

### Study participants

Statistics Norway selected the cohorts by cluster sampling, with schools as the primary unit. In the selection of schools, population density and geography was taken into account. Schools for children with special needs and schools with less than 10 students in either fourth or tenth grade were excluded, leaving 96% of Norwegian fourth and tenth graders in the sampling frame. This national representative cross-sectional examination of randomly selected 9- and 15-year-old children and adolescents was carried out in the “Physical Activity among Norwegian Children Study” in 2005 and 2006. The Regional Committee for Medical Research Ethics and Norwegian Social Science Data Services approved the study. In the study all children in fourth or adolescents from the tenth grade from the consenting schools were invited to participate in physical testing and filling out questionnaire. A total of 2,818 subjects from 40 elementary schools and 23 high schools representing all regions in Norway, were invited. There were no statistical differences between the participants that were included in the analyses compared to those who were excluded regarding mean leisure time physical activity (LTPA) (p = 0.392), proportion of males/females (p = 0.217) or proportion of the three transport modes (p = 0.469). In analyses stratified by age group there was no significant difference in age among children (p = 0.210), whereas among adolescents excluded adolescents were 0.06 years older compared to the included adolescents (p = 0.0068). Furthermore post hoc regression analyses including all possible participants did not produce different estimates for any of the outcomes compared to the main analyses.

### Measurements

All tests were conducted in the school setting by trained personnel and an identical study protocol was used at all schools. Questionnaires were used in order to register mode of transport to school, age, gender and levels of leisure time physical activity. All measurements were collected at the same time as completion of the questionnaire. The data collection was conducted from spring in 2005 till autumn in 2006 (excluding July).

### School transportation

Participants were categorically classified as passive (car/motorcycle or bus/train), walkers and cyclists based on a combination of two questions regarding usual transport mode to and from school. Participants who used the same transportation to and from school were coded accordingly. If information was missing for one direction the participant was coded according to the known transport mode. Participants who walked or cycled one way and used passive transport the other were coded as walkers and cyclists, respectively. Participants who cycled one way and walked the other were, as well as those who did not give any information on transport, coded as missing (n = 13). Information on estimated travel duration was, though available from a categorical question with five options, only crudely assessed and not included in the main analyses.

### LTPA

Physical activity beyond transport to school was defined from a question on weekly hours of non-school related physical activity. Prior to the question on LTPA it was emphasized that only physical activity during leisure time should be summed up in the estimated weekly hours of physical activity. Furthermore it was stressed that all physical activity that increased breathing or perspiration should be included in the estimate. The six options were: “0 hours, 1–2 hours, 3–4 hours, 5–7 hours, 8–10 hours, >11”.

### Anthropometry

Weight and height were measured in light clothing and without shoes. Weight was measured to the nearest 0.1 kg with a digital Seca 770 scale (SECA GmbH, Hamburg, Germany). Height was measured to the nearest 0.1 cm, using wall mounted tapes, with the child standing upright against the wall. BMI was calculated as weight (kg) divided by the height squared (m^2^).

Four skinfold thickness measurements (triceps, biceps, subscapular and suprailiac) were taken using a Harpenden skinfold caliper (John Bull; British Indicators Ltd., West Sussex, England) on the left side of the body according to the criteria described by Lohman et al. [12]. Duplicate measurements were taken for each skinfold, with a third measure taken if the difference between the two measurements differed by ≥ 2 mm, and the two closest measurements were averaged. Inter-rater reliability and intra-rater reliability was assessed from multiple skinfold measurements between and within observers respectively as previously described [13]. Valid measures of CRF was supported by an average HR value of 200.0 (SD = 7.1) whereas high reliability was supported from intra-class (within-observer) and inter-class (between observers) correlation coefficients of 0.95 and 0.84 respectively for skinfold measurements.

### Physical fitness

CFR (i.e. V̇O_2_peak) was assessed in a maximum exercise test on an electronically braked cycle ergometer (Ergomedic 839E, Monark, Varberg, Sweden). Initial and incremental work rates were 20 W for 9-year-olds weighing <30 kg, 25Wfor 9-year-olds weighing ≥30 kg, 40 W for 15-year-old females and 50 W for 15-year-old males. Work rate was increased every third minute until exhaustion while the pedal frequency was maintained at 60–70 rounds per minute. Heart rate (HR) was recorded throughout the test using a HR monitor (Polar Vantage, Polar electro YO, Kempele, Finland) whereas oxygen uptake (V̇O_2_), respiratory exchange ratio (RER) and ventilation were measured every 10 s during the last minutes of the test using a portable MetaMax III X oxygen analyzer (Cortex Biophysics, Leipzig, Germany). We defined V̇O_2_peak as the mean of the three highest consecutive measurements. Every morning the analyzer was calibrated against known gas mixtures, and barometric pressure against values from the local weather station. Criteria of maximal testing were: RER > 0.99 or maximal HR > 185 beats/min and test leader judgement of signs of intense effort (e.g. facial flushing or difficulties in keeping up the pedal frequency). Validation of the metabolic cart and imputation of missing V̇O_2_peak values in 159 participants (due to failure of the V̇O_2_analyzer) was done as previously described [14].

#### Functional strength

Explosive strength of the leg extensors was assessed with a standing jump. The participants stood behind a line with feet slightly apart. They were instructed to perform a two-foot take-off and landing, and to jump as far as possible, landing on both feet without falling backwards. The distance from the take-off line to the nearest point of contact on the landing (back of the heels) was measured, and the better of two attempts was used for analyses [15].

#### Muscle endurance

Dynamic muscle endurance in trunk flexors was measured by sit-ups. The subject was lying face up on the floor with feet held on the ground by the test leader while the knee angle was at 90 degrees. Hands were crossed behind the neck. From the lying position, the participant was instructed to bring their elbows to touch their knees and then to return to the lying position as many times as possible during a 30 second period [15]. Isometric muscle endurance in the trunk extensors was assessed by a modified Biering-Sørensen test [16]. The endurance of the trunk extensor muscles was measured by how many seconds the subject was able to keep the unsupported upper body (from the upper border of the iliac crest) horizontal while placed prone, with the buttocks and legs fixed to a balance pad with the arms folded across the chest.

### Statistical analyses

In order to examine whether the distributions of gender and age group differed according to transport mode the chi squared test was used. Differences in LTPA between transport modes were tested with one-way ANOVAs whereas gender and age differences in distributions of mode of transport were tested with chi squared tests. The associations between mode of transportation to school and physical fitness were tested with multiple linear regression analyses adjusted or stratified for age group and gender (treated as indicator variables) and LTPA (treated as a continuous variable). Interactions between transport mode by gender, age group and LTPA were tested and maintained in the final model if statistically significant (p > 0.05 for interaction). BMI, sum of skin folds, and back extension were due to positive skewnesslog transformed and the means of the transformed data were back-transformed to geometric means. Overall effect of transport mode on outcome variables was tested with likelihood-ratio tests. All analyses were conducted using STATA IC version 11.0 (STATA Corp, College station) with alpha = 0.05.

## Results

A total of 2,299 children and adolescents participated in the study corresponding to a participation rate of 82%. Individuals with missing information on transport mode (n = 464), age (n = 33) and LTPA (n = 497) were excluded. When accounting for overlap between missing in LTPA and transport mode a total of 605 participants were excluded leaving 1694 (i.e. 60% of all invited participants) to be for analysed for associations with physical fitness variables. The distributions of transport modes of the study population are shown in Table 1. Whilst 16.4% of the children passively commuted to school, 77.9% walked and 5.7% cycled. In adolescents, the corresponding values were 34.8%, 42.0%, and 23.3%. Based on chi-squared tests there was no statistical evidence of a difference between genders in relation to mode of commuting in children (p = 0.342) or adolescents (p = 0.109). There was, however, solid statistical evidence of an age difference in distributions of mode of transport to school among both boys (p < 0.0001) and girls (p < 0.0001), which was largely driven by adolescents being more likely to be passive or cyclists compared to children. Mean LTPA across travel modes in boys was 3.3 hours per week compared to 2.8 hours per week in girls. In adolescents, males also tended to be more active averaging 3.5 hours per week compared to 3.3 hours per week in adolescent females. Based on One-way ANOVAs there were no differences in the level of LTPA among children (p = 0.188) using different travelling modes in their commute to school, whereas the means for adolescents were unequal (p = 0.001).

**Table 1:** General characteristics of the studied population (n = 1694)

	**Passive (n = 394)**			**Walking (n = 1092)**			**Cycling (n = 208)**		
	**n**	**Age**	**LTPA**	**n**	**Age**	LTPA^*^	**n**	**Age**	LTPA^*^
Children									
- Boys	89 (15.4%)	9.4 (0.3)	3.1 (1.2)	450 (78.0%)	9.6 (0.4)	3.4 (1.2)	38 (6.6%)	9.5 (0.3)	3.4 (1.2)
- Girls	84 (17.4%)	9.5 (0.3)	2.8 (1.0)	375 (77.8%)	9.6 (0.4)	2.8 (1.1)	23 (4.8%)	9.6 (0.3)	2.7 (0.8)
Adolescents									
- Males	101 (33.0%)	15.5 (0.3)	3.4 (1.4)	123 (40.2%)	15.7 (0.4)	3.6 (1.4)	82 (26.8%)	15.6 (0.4)	3.6 (1.4)
- Females	120 (36.5%)	15.5 (0.4)	3.0 (1.3)	144 (43.8%)	15.6 (0.3)	3.3 (1.3)	65 (19.8%)	15.4 (0.3)	3.5 (1.0)

Data are presented as numbers (percentage within gender and age groups) or means (sd).

^*^LTPA is self-reported hours of physical activity per week beyond school activities.

Table 2 presents mean levels of physical fitness measures by mode of transport in children and adolescents. Except for CRF the unadjusted means for physical fitness measures were in favour of the children walking or cycling to and from school (Table 2). With few exceptions the various physical fitness levels among male and female adolescents were better in walkers compared to passive and better in cyclists compared to walkers (Table 3).

**Table 2:** Descriptive statistics for physical fitness measures by travel mode in boys and girls

	**Boys**						**Girls**					
	**Passive**		**Walking**		**Cycling**		**Passive**		**Walking**		**Cycling**	
	**Mean**	**95% CI**	**Mean**	**95% CI**	**Mean**	**95% CI**	**Mean**	**95% CI**	**Mean**	**95% CI**	**Mean**	**95% CI**
BMI (kg ∙ m^-2^)	17.2	[16.6 ; 17.8]	17.1	[16.9 ; 17.3]	17.1	[16.4 ; 17.8]	17.4	[16.8 ; 18.0]	17.4	[17.2 ; 17.7]	17.2	[16.1 ; 18.5]
Sum of 4 skin folds (mm)	31.3	[28.4 ; 34.6]	31.4	[30.1 ; 32.7]	30.7	[27.0 ; 35.0]	42.2	[38.0 ; 46.8]	42.1	[40.2 ; 44.1]	40.4	[32.4 ; 50.4]
V̇O_2_ peak (mL O_2_/kg/min)	49.4	[47.4 ; 51.6]	48.4	[47.5 ; 49.3]	49.0	[45.9 ; 52.2]	44.1	[42.2 ; 45.9]	42.9	[42.1 ; 43.7]	42.3	[37.9 ; 46.7]
Jump length (cm)	122.1	[117.8 ; 126.4]	124.8	[123.0 ; 126.7]	133.9	[127.5 ; 140.4]	114.7	[110.8 ; 118.7]	117.7	[115.8 ; 119.6]	118.6	[112.4 ; 124.9]
Back extension (s)	45.4	[39.0 ; 52.9]	57.7	[54.3 ; 61.4]	56.3	[45.5 ; 69.5]	54.2	[46.1 ; 63.7]	63.1	[59.5 ; 66.9]	66.6	[47.8 ; 92.6]
Sit ups (numbers)	13.0	[12.0 ; 14.0]	14.0	[13.5 ; 14.4]	14.9	[13.2 ; 16.7]	12.1	[10.9 ; 13.2]	12.5	[12.0 ; 13.0]	12.0	[9.8 ; 14.3]

Due to skewness BMI, Sum of skin folds, and Back extension were log transformed and the means of the logistically transformed data were then back-transformed to a geometric mean. All other are reported as arithmetic means.

**Table 3:** Descriptive statistics for physical fitness measures by travel mode in male and female adolescents

	**Males**						**Females**					
	**Passive**		**Walking**		**Cycling**		**Passive**		**Walking**		**Cycling**	
	**Mean**	**95% CI**	**Mean**	**95% CI**	**Mean**	**95% CI**	**Mean**	**95% CI**	**Mean**	**95% CI**	**Mean**	**95% CI**
BMI (kg ∙ m^-2^)	20.5	[19.9 ; 21.2]	20.4	[20.0 ; 20.9]	19.8	[19.2 ; 20.3]	21.2	[20.7 ; 21.7]	20.8	[20.3 ; 21.2]	21.1	[20.4 ; 21.8]
Sum of 4 skin folds (mm)	34.6	[31.6 ; 38.0]	32.2	[30.1 ; 34.5]	30.4	[28.0 ; 33.0]	59.1	[55.5 ; 63.0]	55.7	[52.5 ; 59.1]	54.4	[49.7 ; 59.5]
V̇O_2_peak (mL O_2_/kg/min)	50.8	[48.6 ; 52.8]	52.4	[50.7 ; 54.1]	55.2	[53.3 ; 57.2]	40.3	[38.9 ; 41.7]	41.4	[40.2 ; 42.5]	43.6	[42.1 ; 45.2]
Jump length (cm)	192.1	[186.6 ; 197.6]	191.7	[187.7 ; 195.7]	199.0	[194.1 ; 204.0]	153.6	[149.2 ; 158.1]	154.3	[150.9 ; 157.7]	162.5	[158.0 ; 167.1]
Back extension (s)	116.7	[109.2 ; 124.7]	113.4	[106.3 ; 120.9]	116.2	[107.3 ; 125.8]	116.6	[107.8 ; 126.1]	121.5	[113.0 ; 130.6]	120.6	[108.6 ; 133.9]
Sit ups (numbers)	22.2	[21.3 ; 23.1]	22.1	[21.4 ; 22.9]	22.6	[21.7 ; 23.6]	18.2	[17.5 ; 18.9]	17.9	[17.3 ; 18.6]	19.8	[18.7 ; 20.9]

Due to skewness BMI, Sum of skin folds, and Back extension were log transformed and the means of the logistically transformed data were then back-transformed to a geometric mean. All other are reported as arithmetic means.

In Table 4 the adjusted estimates of fitness measures are presented. There was no evidence of an association between transport to school and BMI when adjusting for age, gender and LTPA. There was, however, an association between travel mode and sum of 4 skin folds among males but not among females (p = 0.0286 for transport vs. gender interaction). A sum of 4 skin folds of 29.3 mm was statistically lower (p = 0.012) in cycling males compared to walkers (32.8 mm).

**Table 4:** Adjusted estimates in fitness measures between passive transport, walking, and bicycling

	**Passive**	**Walking**	**Cycling**	**Test of transport mode (p-values)**	**Walking vs. passive**	**Cycling vs. passive**	**Cycling vs. walking**	**Number of observations**	**Interaction**
BMI (kg ∙ m^-2^)	18.6	18.4	18.2	0.357	-	-	-	1685	-
Sum of 4 skin folds (mm)	M: 32.2	M: 32.8	M: 29.3		M: 0.597	M: 0.065	M: 0.012	1682	Gender X Transport
	F: 48.9	F: 45.9	F: 47.3	0.043	F: 0.077	F: 0.547	F:0.545		
V̇O_2_peak	M: 50.2	M: 49.4	M: 52.2	0.001	M: 0.319	M: 0.042	M: 0.002	1086	Age X Transport
(mL O_2_/kg/min)	F: 42.3	F:42.8	F:42.2		F: 0.536	F: 0.536	F: 0.559		Gender X Transport
	C: 47.0	C: 45.7	C:45.3		C: 0.083	C: 0.213	C: 0.743		
	A:45.6	A:46.5	A:49.1		A: 0.214	A: 0.000	A: 0.003		
Jump length (cm)	M: 151.1	M: 146.7	M: 161.1	0.000	M: 0.017	M: 0.000	M: 0.000	1678	Gender X Transport
	F: 127.4	F: 133.6	F: 131.7		F: 0.001	F: 0.001	F: 0.472		
Back extension (s)	C:50.3	C: 60.5	C: 60.7	0.015	C: 0.000	C: 0.024	C: 0.964	1673	Age X Transport
	A: 116.4	A: 115.3	A: 115.5		A: 0.855	A: 0.855	A: 0.979		
Sit-ups (numbers)	15.6	15.8	16.5	0.065	-	-	-	1672	-

Multiple regression analysis, adjustedor stratified for age, gender, and LTPA level. In case of interaction the analysis was stratified by the interaction term. Estimates based on average age, sex and LTPA.

M:male (boys and adolescent males).

F: female (girls and adolescent females).

C. = children (9 year old).

A. = adolescents (15 year old).

note: BMI and S4SF, Back extension was log transformed in order to meet model requirements.

There was statistical evidence that the association between CRF and school transport was modified by both age (p = 0.0039) and gender(p = 0.0290). Among male cyclists mean CRF was 52.2 mL O_2_/kg/min, which was higher than observed in both walkers (49.4 mL O_2_/kg/min;p = 0.002) and passive commuters (50.2 mL O_2_/kg/min;p = 0.042). Adolescent cyclists had significantly better CRF (49.1 mL O_2_/kg/min) than passive (beta: 45.6, p = 0.000) and walkers (beta: 46.5, p = 0.003). CRF did not differ between walkers and passive commuters neither when comparing across gender nor when comparing across age groups.

Functional strength measures were likewise subject to gender vs. transport interaction. Longer jump length was, despite of cycling vs. walking females and walking vs. passive males, observed in all walkers vs. passive and cyclists vs. passive comparisons. Muscle endurance measured as back extension duration in seconds was significantly longer in children walking (beta: 60.5, p = 0.000) and cycling (60.7, p = 0.024) to/from school compared to passive. There were no statistical difference between cycling and walking children nor did adolescent estimates of back extension differ across transport modes.

## Discussion

### Anthropometrics

This study demonstrates that that males cycling to school had lower sum of skin folds than adolescents walking to school. This was evident from a statistically significant lower sum of skin folds observed in males cycling compared to walkers (p = 0.012) and borderline significantly lower when compared to passive commuters (p = 0.065). Lower sum of skin folds could be caused by higher intensity and thus higher energy expenditure among cyclists compared to walkers, which has been observed in adult commuters [11]. Higher energy expenditure among cyclists can, however, only be established if travel duration was equal or longer compared to walkers. In the present study crude post hoc analyses of self-reported estimates of travel duration to school showed somewhat longer travelling time among walkers compared in relation to cyclists. Thus, based on the available data, it cannot be ruled out that a shorter travel duration among cyclists may have counteracted the possible higher energy expenditure during and after (due to higher excess post exercise oxygen consumption) commuting. Nevertheless the findings in this study contribute to the conception that there is a greater effect on weight status of cycling compared to walking to school, although the previous reported lower BMI among cyclists compared to passive commuters [10] was not confirmed. An inferior effect of walking could explain why the anticipated lower BMI among walkers compared to passive commuters was not detected. Further, relatively few cyclists could explain absence of an association with BMI in the present study. The latter is supported from previous studies which in spite of twice as many included cyclists as in this study did not detect an association with BMI [8,9].

### Cardiorespiratory and muscular fitness

Higher CRF in adolescents and male cyclists compared to walkers and passive commuters were observed. It is possible that the expected higher mean CRF among cycling children and females has been subject to lack of statistical power because of few female cyclists, especially in the youngest group of participants. CRF in walkers was, albeit quantitative strongly represented, not different from passive commuters. These findings are in line with a previous observational study by Cooper et al. [9], who adjusted for accelerometer assessed LTPA and with the study by Andersen et al. [8] who solely adjusted for gender. Thus, from the available data it seems plausible that walking to school, possible due to low intensity, is inadequate with respect to affecting maximal CRF. This is indirectly supported by the notion that cycling, but not walking, to work is associated with intensities consistent with CRF adaptations [17]. Yet, no study comparing travel intensities to school between walkers and cyclists has been conducted. One study, however, reported average cycling intensity to school at about 74% of maximal HR frequency [18], which indeed would make CRF adaptations possible. In a public health perspective cycling to school appear to be relevant since high CRF is strongly associated to clustering of cardiovascular disease risk factors [19].

Higher isometric muscle endurance in the back extensors of the children cycling and walking compared to passive commuters might be considered relevant in the prevention of back pain since children with isometric muscle endurance are less likely to report back pain [20].

Performance in sit-ups (abdominal flexor strength) was not different across travel modes whereas better dynamic muscle strength among cyclists was observed. The latter may be associated to higher bone mineral density [21] if not counteracted by, yet un-established, but possible adverse effects of cycling on bone health [22].

Considering physiological strain during cycling these muscular specific adaptations are expected and indirectly support that the differences observed between travel modes in fact is due to active transportation and not because of selection bias.

### Strengths and limitations

A major strength of this study was the large population-based sample with a high participation rate. Furthermore, direct measurements of V̇O_2_ peak as well as measurement carried out by skilled test personnel are advantageous. There are several limitations which should be considered when interpreting the findings of this study. Due to the cross*-*sectional nature of the study design, causal inferences cannot be made. Another major limitation of the study includes the use of self-reported measure of LTPA. In fact available objective accelerometer determined data on LTPA was neglected. The reason for this was that adjustment for accelerometer assessed LTPA would involve adjustment for the exposure of interest and conceal a potential effect of active transport to school. Furthermore, it would be problematic that the degree of adjustment of exposure would be much greater in walkers compared to cyclists because accelerometer are limited by their inability to capture cycling as they underestimate intensity during cycling with up to 90% [23]. Assuming that the amount of accelerometer counts picked up during cycling is negligible, an analysis with adjustment for LTPA based on objectively measured accelerometer counts; instead of self-report should be a valid comparison between estimates for passive commuting and cycling. Based on such a post hoc analysis for V̇O_2_peak similar results were found when adjusting for self-reported LTPA. Thus, there is indirect evidence that the present results were not subject to residual LPTA confounding. However, we cannot rule out that the results were subject to residual confounding from socioeconomic status, pubertal status, diet or school distance.

The prevalence of cycling to school was much lower than observed in Danish studies where approximately 38% of the children [9] and 62% of the adolescents [10] cycle to school. Besides from cultural and infrastructural differences a relative strenuous and dark winter (due to few hours of daylight especially in the northern part of Norway) could be the reason for this. In fact a previous study reported that there is large seasonal variance in especially cycling to school in Norway [24] with a very low proportion (i.e. 3%) of cyclists during winter compared to fall and spring (≈50%). It is likely that children measured in winter and categorized as passive or walking had been cycling to school months earlier during the fall and likewise and vice versa for participants measured during spring. This would relative to the degree of inertia of changes in physical fitness outcomes obscure the present results.

More research on the effectiveness of various commuter cycling initiatives is needed in order to be able to establish a cost beneficial primary prevention strategy. Furthermore future studies should investigate the associations between transport mode to school and hematological risk factors, while to a higher degree taking into account potential sources of residual confounding.

## Conclusions

In conclusion the lower sum of skin folds observed in male cyclists indicated that cycling to school could be an important supplement in the prevention of adverse weight gain. Cycling but not walking was associated with better CRF compared to passive transport to school. Thus, from a public health perspective promoting cycling to school may be possible strategy in the prevention of cardiovascular diseases. Active commuters showed better back muscle endurance as compared to passive commuters thus walking and cycling could be relevant in the prevention of back pain. Standing jump length reflecting dynamic extensor muscle strength was better in male cyclists compared to walkers and passive, whereas walking generally was not associated with higher muscular performance compared to passive. Better dynamic muscle strength among cyclists may be associated to higher bone mineral density.

Finally, active commuting, especially cycling, seem to be associated with a favourable body composition, better cardiorespiratory and muscular fitness as compared to passive commuting.

### Consent

We obtained written, informed consent from the child's parent or legal guardian after they were given, in writing, a full explanation of the aims of the study and its possible hazards, discomfort, and inconvenience.

## Abbreviations

LTPA: Leisure time physical activity; HR: Heart rate; V̇O_2_: Oxygen uptake; SD: Standard deviation; RER: Respiratory exchange ratio; CRF: Cardiorespiratory fitness; BMI: Body mass index.

## Competing interests

All authors have completed the ICMJE uniform disclosure form at http://www.icmje.org/coi_disclosure.pdf (available on request from the corresponding author) and declare: no support from any organization for the submitted work.

## Authors’ contributions

LØ analyzed and interpreted data, drafted the manuscript, and is guarantor. LBA and SAA designed the study interpreted data and revised the manuscript. EK and JSH collected, interpreted the data and revised the manuscript. All authors approved the submitted version of the manuscript.
